# Eigen decomposition expedites longitudinal genome-wide association studies for milk production traits in Chinese Holstein

**DOI:** 10.1186/s12711-018-0383-0

**Published:** 2018-03-26

**Authors:** Chao Ning, Dan Wang, Xianrui Zheng, Qin Zhang, Shengli Zhang, Raphael Mrode, Jian-Feng Liu

**Affiliations:** 1 National Engineering Laboratory for Animal Breeding, Key Laboratory of Animal Genetics, Breeding and Reproduction, Ministry of Agriculture, College of Animal Science and Technology, China Agricultural University, Beijing, 100193 China; 2 Animal Biosciences, International Livestock Research Institute, Nairobi, 00100 Kenya

## Abstract

**Background:**

Pseudo-phenotypes, such as 305-day yields, estimated breeding values or deregressed proofs, are usually used as response variables for genome-wide association studies (GWAS) of milk production traits in dairy cattle. Computational inefficiency challenges the direct use of test-day records for longitudinal GWAS with large datasets.

**Results:**

We propose a rapid longitudinal GWAS method that is based on a random regression model. Our method uses Eigen decomposition of the phenotypic covariance matrix to rotate the data, thereby transforming the complex mixed linear model into weighted least squares analysis. We performed a simulation study that showed that our method can control type I errors well and has higher power than a longitudinal GWAS method that does not include time-varied additive genetic effects. We also applied our method to the analysis of milk production traits in the first three parities of 6711 Chinese Holstein cows. The analysis for each trait was completed within 1 day with known variances. In total, we located 84 significant single nucleotide polymorphisms (SNPs) of which 65 were within previously reported quantitative trait loci (QTL) regions.

**Conclusions:**

Our rapid method can control type I errors in the analysis of longitudinal data and can be applied to other longitudinal traits. We detected QTL that were for the most part similar to those reported in a previous study in Chinese Holstein. Moreover, six additional SNPs for fat percentage and 13 SNPs for protein percentage were identified by our method. These additional 19 SNPs could be new candidate quantitative trait nucleotides for milk production traits in Chinese Holstein.

**Electronic supplementary material:**

The online version of this article (10.1186/s12711-018-0383-0) contains supplementary material, which is available to authorized users.

## Background

Complex traits that require observations over multiple time points for the same individual are called longitudinal traits. A typical example are milk production traits in dairy cattle, which are usually measured once every month during the lactation period. Analysis of such data provides the opportunity to investigate the genetic control of dynamic processes [1].

In genome-wide associate studies (GWAS) of milk production traits in dairy cattle, multiple phenotypic measurements for each individual are transformed into a single measure, such as, 305-day yield, an estimated breeding value (EBV) or a deregressed proof (DRP). Then, these pseudo-phenotypes are used as response variables in the GWAS statistical model [2–4]. GWAS using raw phenotypes are considered as the “gold standard” [5], and pseudo-phenotypes can result in reduced power or even inflate false positive rates (FPR) for the detection of quantitative trait nucleotides (QTN) [6]. Furthermore, longitudinal GWAS that directly uses multiple phenotypic measurements can model time-dependent QTN effects. Nevertheless, longitudinal GWAS has seldom received much attention.

In our previous study [7], we applied random regression models to longitudinal GWAS. Simulation and real data studies showed that the proposed methods based on longitudinal phenotypes outperformed GWAS methods that are based on pseudo-phenotypes, both regarding control of FPR and power of QTN detection. However, this method was computationally inefficient as the dimension of the mixed model equation increased and as the number of variance components to be estimated increased compared with the non-longitudinal GWAS model. It took about 10 days for the analysis of about 6000 individuals (about 50,000 phenotypic records) genotyped with 70 K SNPs even when a sparse pedigree-derived relationship matrix was used. The analysis could not be performed when a dense marker-based kinship matrix was used to reflect individual genetic relationships.

Eigen decomposition of the kinship matrix is a useful tool that can be used to speed up GWAS analysis. It was first proposed by Kang et al. [8], and later extended by Lippert et al. [9], Zhou and Stephens [10] and Xu [11]. Here, we applied the algorithm to longitudinal GWAS. The relationships between individuals and between continuous measures were removed through a transformation procedure, which involved rotation of the data and then the complex random regression model was transformed into a weighted least squares model for each SNP test.

A series of simulation studies was carried out to evaluate the performance of our method. GWAS for milk production traits in Chinese Holstein were previously performed by Jiang et al. [3] using EBV estimated from the first three parities. In this paper, our aim was to further validate the results from the new method through the analysis of actual observed test-day phenotypes for milk yield (MY), fat percentage (FP) and protein percentage (PP) in the first three parities in a larger Chinese Holstein population. In addition, we validated the results of the new method by comparing the detected quantitative trait loci (QTL) for MY, FP and PP with the tens of thousands QTL from previous publications as stored in the QTLdb database [12].

## Methods

### Statistical analyses

The proposed method is based on a random regression model [13], and an additional fixed regression term was incorporated to explain the time-dependent SNP effect:

1 $$y_{ijn} \left( t \right) = \mu_{i} \left( t \right) + htd_{j} + x_{nm} SNP_{m} \left( t \right) + a_{n} \left( t \right) + pe_{n} \left( t \right) + e_{nt} ,$$

where $y_{ijn} \left( t \right)$ is the phenotypic value of individual $n$ at time point $t$; $\mu_{i} \left( t \right)$ is the overall mean for the $i$-th group at time $t$ and cows calving in the same season and of similar age are in the same group; $htd_{j}$ is herd-test-date (HTD) effect; $x_{nm}$ is a genotype indicator for the $m$-th SNP which is assigned 0, 1 and 2 for genotype $aa$, $Aa$ and $AA$, respectively; $SNP_{m} \left( t \right)$ represents the time-dependent additive SNP effect; $a_{n} \left( t \right)$ and $pe_{n} \left( t \right)$ are the time-dependent additive genetic effect and permanent environmental effect, respectively for individual $n$; $e_{nt}$ is the time-independent random residual for each measurement of individual $n$ at time $t$. Here, $\mu_{i} \left( t \right)$, $SNP_{m} \left( t \right)$, $a_{n} \left( t \right)$ and $pe_{n} \left( t \right)$ can be denoted as a set of Legendre polynomials or any other curve parameter (such as Wilmink polynomials [14]).

2 $$\begin{aligned} \mu_{i} \left( t \right) & = \mathop \sum \limits_{k = 0}^{{nf_{1} }} \beta_{ik} \varphi_{k} \left( t \right),SNP_{m} \left( t \right) \\ & = \mathop \sum \limits_{k = 0}^{{nf_{2} }} \alpha_{mk} \varphi_{k} \left( t \right),a_{n} \left( t \right) \\ & = \mathop \sum \limits_{k = 0}^{{nr_{1} }} u_{nk} \varphi_{k} \left( t \right),pe_{n} \left( t \right) \\ & = \mathop \sum \limits_{k = 0}^{{nr_{2} }} p_{nk} \varphi_{k} (t), \\ \end{aligned}$$

where $nf_{1}$, $nf_{2}$, $nr_{1}$ and $nr_{2}$ are the orders of the corresponding polynomials; $\varphi_{k} \left( t \right)$ is the value of the $k$-th polynomial at time $t$; $\beta_{ik}$ and $\alpha_{mk}$ is the $k$-th fixed regression coefficient for time-dependent mean and additive SNP effect, respectively; $u_{nk}$ and $p_{nk}$ are the $k$-th random regression coefficients for additive genetic effect and permanent environmental effect of individual $n$.

The model in matrix notation is:

3 $${\mathbf{y}} = {\mathbf{Xb}} + {\mathbf{Qu}} + {\mathbf{Zp}} + {\mathbf{e}} ,$$

where ${\mathbf{y}}$ is the vector of test-day records; ${\mathbf{b}}$ is the vector of solutions for HTD and fixed regressions; ${\mathbf{u}}$ and ${\mathbf{p}}$ are the vectors of random regression coefficients for additive genetic effects and permanent environmental effects, respectively; ${\mathbf{X}}$, ${\mathbf{Q}}$ and ${\mathbf{Z}}$ are the corresponding design matrices; ${\mathbf{e}}$ is the vector of random residuals. It is assumed that:

4 $${\mathbf{u}} \sim N({\mathbf{0}},{\mathbf{K}} \otimes {\mathbf{G}}),\,{\mathbf{p}} \sim N({\mathbf{0}},{\mathbf{I}} \otimes {\mathbf{P}}),\,{\rm and}\,{\mathbf{e}} \sim N\left( {{\mathbf{0}},{\mathbf{I}}\sigma_{e}^{2} } \right)$$

where, ${\mathbf{K}}$ is the marker-derived relationship matrix; ${\mathbf{I}}$ is the identity matrix; $\otimes$ is the Kronecker product; ${\mathbf{G}}$ is the variance–covariance matrix for random regression coefficients of additive polygenic effects; ${\mathbf{P}}$ is the variance–covariance matrix of random regression coefficients for permanent environmental effects; $\sigma_{e}^{2}$ is the residual variance. ${\mathbf{K}}$ was built with the method of VanRaden [15] as follows:

$${\mathbf{K}} = {\mathbf{MM}}'/2\mathop \sum \nolimits p_{i} (1 - p_{i} ),$$

where $p_{i}$ is the second allele at locus $i$, and the $i$-th column of matrix ${\mathbf{M}}$ is:

$${\mathbf{m}}_{i} = \left\{ {\begin{array}{*{20}c} {2 - 2p_{i} } & {AA} \\ {1 - 2p_{i} } & {Aa} \\ {0 - 2p_{i} } & {aa} \\ \end{array} } \right..$$

Similar to the study of Kang et al. [16] and Zhang et al. [17], we initially estimated the variance components without including the SNP effect in the model, and then these estimates were applied in the model that examines whole-genome association effects of SNPs. With known variance components, the time-dependent additive genetic, permanent and phenotypic variances can be expressed as:

5 $$\begin{aligned} add\left( t \right) &= [\varphi_{0} \left( t \right), \ldots , \varphi_{k} \left( t \right), \ldots , \varphi_{{nr_{1} }} \left( t \right)] \\&\times {\hat{\mathbf{G}}}[\varphi_{0} \left( t \right), \ldots , \varphi_{k} \left( t \right), \ldots , \varphi_{{nr_{1} }} \left( t \right)]^{T},\\&perm\left( t \right) = [\varphi_{0} \left( t \right), \ldots , \varphi_{k} \left( t \right), \ldots , \varphi_{{nr_{2} }} \left( t \right)]\\ &\times {\hat{\mathbf{P}}}[\varphi_{0} \left( t \right), \ldots , \varphi_{k} \left( t \right), \ldots , \varphi_{{nr_{2} }} \left( t \right)]^{T},\\ &\,{\text{and}}\,phe \left( t \right) = add\left( t \right) + perm\left( t \right) + \hat{\sigma }_{e}^{2} .\end{aligned}$$

The phenotypic variance–covariance matrix is:

6 $${\text{var}}\left( {\mathbf{y}} \right) = {\mathbf{Q}}\left( {{\mathbf{K}} \otimes {\hat{\mathbf{G}}}} \right){\mathbf{Q}}^{T} + {\mathbf{Z}}\left( {{\mathbf{I}} \otimes {\hat{\mathbf{P}}}} \right){\mathbf{Z}}^{T} + {\mathbf{I}}\hat{\sigma }_{e}^{2} ,$$

where, ${\mathbf{Q}}\left( {{\mathbf{K}} \otimes {\hat{\mathbf{G}}}} \right){\mathbf{Q}}^{T}$ reflects the covariance relationship between individuals, while ${\mathbf{Z}}\left( {{\mathbf{I}} \otimes {\hat{\mathbf{P}}}} \right){\mathbf{Z}}^{T}$ reflects the covariance relationship between successive records of each individual. Now, we show how to transform these covariance matrices to diagonal matrices using Eigen decomposition.

We define ${\mathbf{W}} = {\mathbf{Q}}\left( {{\mathbf{K}} \otimes {\hat{\mathbf{G}}}} \right){\mathbf{Q}}^{T} + {\mathbf{Z}}({\mathbf{I}} \otimes {\hat{\mathbf{P}}}){\mathbf{Z}}^{T}$ and the Eigen decomposition for ${\mathbf{W}}$ is ${\mathbf{W}} = {\mathbf{UDU}}^{T}$, where ${\mathbf{D}}$ is a diagonal matrix containing the eigenvalues and ${\mathbf{U}}$ is the matrix of eigenvectors in the order of the corresponding eigenvalues. We rotate Eq. (3) with ${\mathbf{U}}^{T}$, and the mixed model can be rewritten as:

7 $${\mathbf{U}}^{T} {\mathbf{y}} = {\mathbf{U}}^{T} {\mathbf{Xb}} + {\mathbf{U}}^{T} ({\mathbf{Qu}} + {\mathbf{Zp}} + {\mathbf{e}}) ,$$

8 $${\mathbf{y}}^{*} = {\mathbf{X}}^{*} {\mathbf{b}} + {\mathbf{e}}^{*} ,$$

where ${\mathbf{y}}^{*} = {\mathbf{U}}^{T} {\mathbf{y}}$, ${\mathbf{X}}^{*} = {\mathbf{U}}^{T} {\mathbf{X}}$ and ${\mathbf{e}}^{*} = {\mathbf{U}}^{T} ({\mathbf{Qu}} + {\mathbf{Zp}} + {\mathbf{e}})$. The covariance matrix of rotated phenotypic values is:

9 $$\begin{aligned} {\text{var}}\left( {{\mathbf{y}}^{*} } \right) & = {\text{var}}\left( {{\mathbf{e}}^{*} } \right) \\ & = {\mathbf{U}}^{T} \left( {{\mathbf{Q}}\left( {{\mathbf{K}} \otimes {\hat{\mathbf{G}}}} \right){\mathbf{Q}}^{T} + {\mathbf{Z}}\left( {{\mathbf{I}} \otimes {\hat{\mathbf{P}}}} \right){\mathbf{Z}}^{T} + {\mathbf{I}}\hat{\sigma }_{e}^{2} } \right){\mathbf{U}} \\ & = {\mathbf{U}}^{T} {\mathbf{Q}}\left( {{\mathbf{K}} \otimes {\hat{\mathbf{G}}}} \right){\mathbf{Q}}^{T} + {\mathbf{Z}}\left( {{\mathbf{I}} \otimes {\hat{\mathbf{P}}}} \right){\mathbf{Z}}^{T} ){\mathbf{U}} + {\mathbf{U}}^{T} {\mathbf{U}}\hat{\sigma }_{e}^{2} \\ & = {\mathbf{U}}^{T} {\mathbf{WU}} + {\mathbf{U}}^{T} {\mathbf{U}}\hat{\sigma }_{e}^{2} . \\ \end{aligned}$$

It should be noted that ${\mathbf{W}} = {\mathbf{UDU}}^{T}$ and ${\mathbf{UU}}^{T} = {\mathbf{I}}$,

then

10 $${\text{var}}\left( {{\mathbf{y}}^{*} } \right) = {\text{var}}\left( {{\mathbf{e}}^{*} } \right) = {\mathbf{U}}^{T} {\mathbf{UDU}}^{T} {\mathbf{U}} + {\mathbf{U}}^{T} {\mathbf{U}}\hat{\sigma }_{e}^{2} = {\mathbf{D}} + {\mathbf{I}}\hat{\sigma }_{e}^{2} .$$

Let ${\mathbf{S}} = {\mathbf{D}} + {\mathbf{I}}\hat{\sigma }_{e}^{2}$ with ${\mathbf{S}}$ being a diagonal matrix. Then, Eq. (8) can be solved by weighted least squares:

11 $${\hat{\mathbf{b}}} = ({\mathbf{X}}^{*T} {\mathbf{S}}^{ - 1} {\mathbf{X}}^{*} )^{ - 1} {\mathbf{X}}^{*T} {\mathbf{S}}^{ - 1} {\mathbf{y}}^{*} ,$$

12 $${\text{var}}\left( {{\hat{\mathbf{b}}}} \right) = ({\mathbf{X}}^{*T} {\mathbf{S}}^{ - 1} {\mathbf{X}}^{*} )^{ - 1} .$$

From Eqs. (11) and (12), ${\hat{\mathbf{b}}}_{SNP}$ is the estimated value of fixed regression coefficients for additive SNP effects, and ${\text{var}}({\hat{\mathbf{b}}}_{SNP} )$ the corresponding variance. Therefore, the Wald Chi squared test for time-dependent SNP effect is:

13 $${\hat{\mathbf{b}}}^{T} \left[var\left( {{\hat{\mathbf{b}}}_{SNP} } \right)\right]^{ - 1} {\hat{\mathbf{b}}} \sim \chi^{2} (nf_{2} + 1) .$$

The Bonferroni correction is used to control the rate of false-positive rates. Therefore, the threshold for genome-wide significance is 0.05/ ${\text{M}}$, where ${\text{M}}$ is the effective number of SNPs which is calculated by PLINK software with the command “–indep-pairwise 50 5 0.5”. This PLINK command produces a pruned subset of SNPs that are in approximate linkage equilibrium (LD) with each other through the application of the following steps: (1) it calculates LD between each pair of SNPs within a window of 50 SNPs; (2) it removes one SNP of a pair that has a LD greater than 0.2 until no such pairs remain; and (3) it shifts the window five SNPs forward and then repeats the procedure across the whole genome.

### Data

**Fig. 1:**
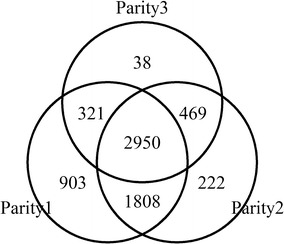
Number of dairy cows for the first three parities used in longitudinal GWAS

The original study on the population of Chinese Holsteins consisted of 9615 genotyped animals and the detailed genotypic information is described in Ning et al. [7]. Three quantitative traits consisting of MY, FP and PP in the first three parities were analyzed in this study. The number of cows with more than five test day records in the three parities is in Fig. 1. The SNPs with a minor allele frequency (MAF) lower than 0.03 and those that failed the Hardy–Weinberg equilibrium (HWE) test (*P* value < 10–6) were removed for each parity, resulting in 71,527 common SNPs for the subsequent longitudinal GWAS analyses.

### Simulation

**Fig. 2:**
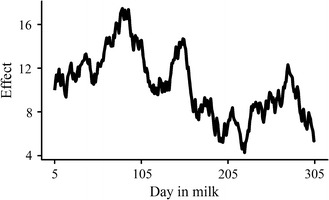
Time-varied simulated additive genetic effect for random SNPs

We performed extensive simulations to compare systematically the performance of the models using two different curve parameters (Legendre polynomials and Wilmink polynomials). In order to evaluate our method, another model, developed by Das et al. [18] that deals with human functional traits, was also included in our study. The latter model did not include time-varied additive polygenic effects, which was expected to inflate type I errors for the cow group with population structure and cryptic relatedness. Our simulation was similar to that described in the study of Yu et al. [19]. In order to assess the null distribution of different models, 1000 random SNPs were tested for MY, FP and PP in the first parities. Under the expectation that random SNPs are unlinked to polymorphisms controlling these traits, the cumulative *P*-value distribution follows a uniform distribution. We tested a simulation of statistical power by adding an additional time-varied genetic effect to observed phenotypes. The genetic effect for the first day (Day 5) was set to 10.0, and the genetic effect for the day after was simulated by adding a value sampled from a uniform distribution of $U( - 1,1)$. The simulated time-varied genetic effect is illustrated in Fig. 2. The cumulative effect was scaled to $k$ = 0.04, 0.08, 0.12, 0.16 and 0.20 times the phenotypic standard deviation. The percentage ($\pi$) of the total phenotypic variation explained by this genetic effect can be estimated as:

$$\pi = \frac{{2p(1 - p)k^{2} }}{{1 + 2p(1 - p)k^{2} }},$$

where $p$ is the allele frequency of each SNP. The genetic effect was assigned to 1000 random SNPs, one at a time, and each model was run to determine whether the effect could be detected with the empirical threshold (the 5th percentile of a null distribution).

### Implementation of the software

The variance components were estimated using the DMU package (http://dmu.agrsci.dk/DMU/) and the longitudinal GWAS analyses were performed by a custom written Python script available at https://github.com/chaoning/longEigen.

## Results

### Simulation studies

**Fig. 3:**
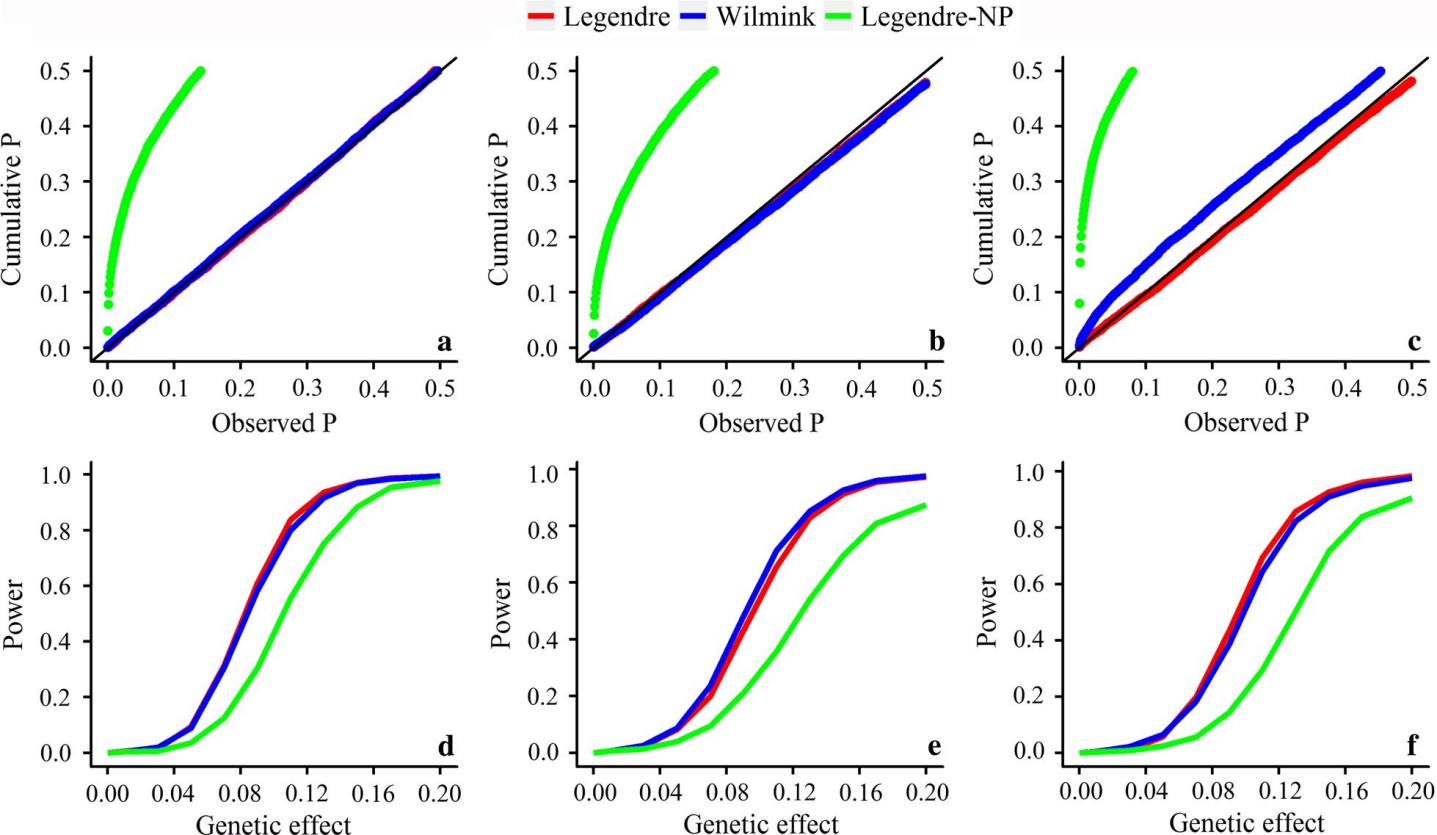
Cumulative *P*-value distributions and empirical power of different models. **a**–**c** Cumulative *P*-value distributions using random SNPs for MY, FP and PP. Under the assumption that random SNPs are unlinked to these traits, models that appropriately control for type I errors should show a uniform distribution of *P* values. **d**–**f** Empirical power of different models for MY, FP and PP. The empirical threshold was determined as the 5th percentile of a null distribution

The cumulative *P*-value distribution of random SNPs are in Fig. 3a–c. Overall, the Legendre model showed good control of type I errors. The Legendre-NP model (Legendre model that does not include time-varied polygenic effects) should result in large type I errors. For PP, the Wilmink model had a convergence problem in the estimation of variance components, which resulted in a type I error with non-convergent variance component. The empirical power in Fig. 3d–f showed that Legendre and Wilmink models had similar adjusted power, but it greatly exceeded that of the Legendre-NP model.

### Time-dependent variances

**Fig. 4:**
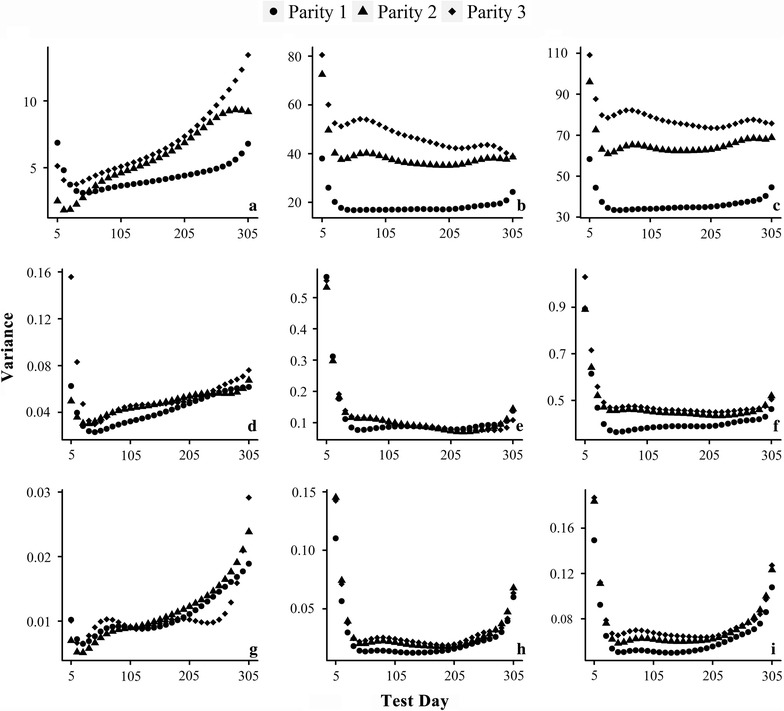
Time-dependent additive genetic (left), permanent environmental (middle) and phenotypic (right) variances for MY (top), FP (middle) and PP (bottom) across the lactation period

The additive genetic, permanent environmental and phenotypic variances across the lactation period for the first three parities are in Fig. 4. Additive genetic variances were higher at the beginning, then dropped sharply to the lowest point before day 50, and increased during the remaining lactation. Permanent environment and phenotypic variances share very similar pattern changes, since the former is the major component of the latter. These variances were higher at the beginning and decreased thereafter, and then a relatively long plateau occurred with the exception of a slight increase at the end of lactation for FP and PP. Furthermore, the variances increased from parity to parity.

### Significant SNPs

**Fig. 5:**
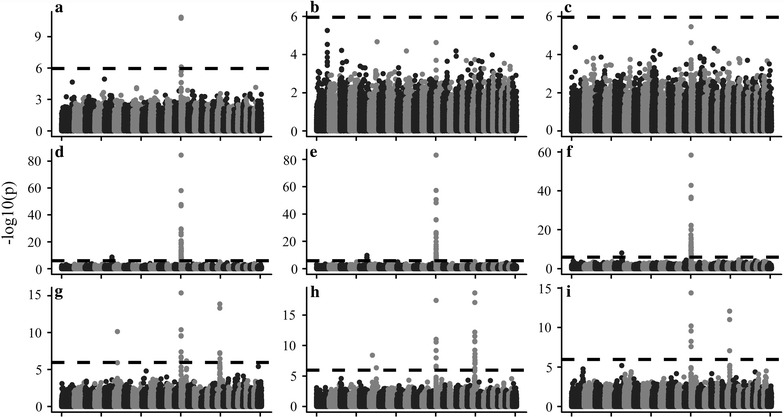
Manhattan plots of –log_10_(*P*) for MY (**a**, **b**, **c**), FP (**d**, **e**, **f**) and PP (**g**, **h**, **i**) of the first (**a**, **d**, **g**), second (**b**, **e**, **h**) and third (**c**, **f**, **i**) parities

We analyzed the MY, FP and PP for three parities with the Legendre model since it is able to control type I errors and showed good convergence in the estimation of variances for all traits. After running the PLINK software with the command “–indep-pairwise 50 5 0.5”, 45,573 pruned SNPs in approximate linkage equilibrium with each other were retained. Therefore, the significant threshold was 0.05/45,573 = 1.097E − 6 after Bonferroni correction. In total, we detected 84 genome-wide significant SNPs by our method. The Manhattan plots of $- \log_{10} (p)$ in Fig. 5 reveals four peaks on respectively chromosomes 5, 6, 14 and 20, which influence milk production traits in Chinese Holstein. The peak on chromosome 14 influences all three traits, and the well-known *DGAT1* (*diacylglycerol O*-*acyltransferase 1*) gene, reported to be a major gene affecting milk production traits [20], is located within this region. The detailed information of all significant SNPs for milk yield (MY), fat percentage (FP) and protein percentage (PP) is provided in Table S1 [see Additional file 1: Table S1], including their positions on the genome, *P* values for the three parities, known genes within a 100-kb region around the SNP, and QTL ID for nearest QTL. We obtained QTL information from the animal QTLdb [12] and only focused on the QTL that were identified by the association analysis method and had relatively narrow and accurate estimated intervals. The key findings are summarized as follows.

For milk yield (MY), we found three significant SNPs for the first parity and none for the second and third parities. One of these SNPs is located within the *DGAT1* gene and all significant SNPs are within the reported QTL for MY.

For fat percentage (FP), 65 significant SNPs were identified, of which, 64 showed association in the first parity, while an additional SNP was identified for both the second and third parities. Furthermore, five of the 65 SNPs are located between 93.48 and 94.27 Mb on chromosome 5 and the others are within a 4.68-Mb segment (1.40–6.10 Mb) on chromosome 14. We mapped these significant SNPs to the animal QTLdb [12] and discovered that 59 were located in the reported QTL for FP. The remaining SNPs were 16 to 107 kb away from the nearest reported QTL.

For protein percentage (PP), we identified 28 SNPs, of which 27 were identified for the second parity and an additional SNP identified for the first parity. Furthermore, nine SNPs are located between 1.65 and 2.06 Mb on chromosome 14 and 15 SNPs are clustered within a region between 31.93 and 34.82 Mb on chromosome 20. We found that 15 of the significant SNPs were within the regions that were previously reported to harbor QTL for PP, while the 13 remaining SNPs are 4 to 1.724 kb away from the nearest QTL.

In total, 65 of the 84 significant SNPs were within reported QTL regions. In order to evaluate whether the significant SNPs were located in a QTL region by chance, we conducted an enrichment study similar to the odds ratio method that is commonly used in case–control studies. According to the reported QTL for MY, FP and PP, the bovine whole genome was divided into QTL regions and non-QTL regions. The QTL regions occupied 6.25% of the genome (168,709,938 bp). Thus, among the 84 random SNPs, about five (84*6.25%) were located in QTL regions by chance. The enrichment coefficient was calculated as the number of significant SNPs per bp in a QTL region versus the number of significant SNPs per bp in a non-QTL region. The enrichment coefficient is 1 if the significant SNPs were randomly located across the whole genome. In our study, the enrichment coefficient was equal to 54.7 (*p*-value < 2.2e-16).

**Fig. 6:**
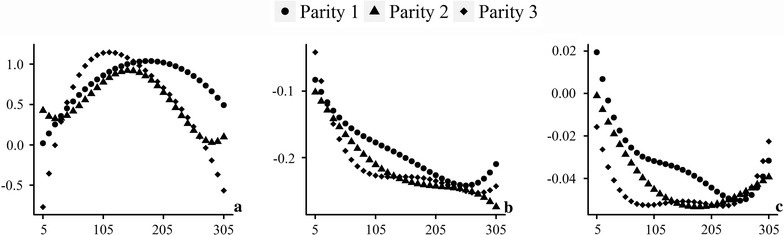
The time-varied effects of SNP ARS-BFGL-NGS-4939 (within the *DGAT1* region) for MY (left), FP (middle), and PP (right) in the first three parities

The time-varied effects of SNP ARS-BFGL-NGS-4939 (within the *DGAT1* gene region) for three traits in the first three parities are in Fig. 6. Alleles of this SNP are *G* and *C*, with frequencies of 0.209 and 0.791, respectively. In our study, genotypes *GG*, *GC* and *CC* were coded 0, 1 and 2, respectively. In general, the time-varied effects for SNP ARS-BFGL-NGS-4939 shared very similar changing patterns in the three different parities. The genotype *CC* with the highest frequency had a greater genotypic value compared to genotypes *GC* and *CC* for the MY trait, whereas the opposite was observed for the FP and PP traits.

### Computational efficiency

Eigen decomposition could expedite longitudinal GWAS in theory and practice. From Eqs. (11) and (12), we can see that matrix multiplication between the inverse of the phenotypic covariance matrix (${\mathbf{S}}^{ - 1}$) and the design matrix for fixed effects (${\mathbf{X}}$) has the largest impact on computational efficiency. Eigen decomposition can rotate the dense phenotypic covariance matrix into a diagonal matrix, and theoretical computational time can be improved approximately $N$ times, where $N$ is the total number of observed records. In practice, our method could complete the analysis in 1 day with known variance components on a Linux computer of 24 cores. However, only 50 SNP tests could be completed under these conditions.

## Discussion

In this study, we performed longitudinal GWAS for three milk production traits in the first three parities of Chinese Holstein. To our knowledge, this is the first GWAS for milk production traits using test-day records as response variable. In addition, a marker-derived genomic relationship matrix was included to reflect the relationship between individuals. Compared with the pedigree-based relationship matrix, which was used in previous studies [3, 4], the marker-derived genomic relationship matrix is more precise for specifying the actual relationships between individuals. For example, differences among full siblings cannot be distinguished with the pedigree-based compared to the marker-derived genomic relationship matrix. However, a dense marker-derived matrix usually results in a strong increase in computational burden. Several effective methods [8–10, 16] have been proposed to overcome this problem for non-longitudinal traits, *i.e.*, one record per individual. Here, we expanded the Eigen decomposition technique to longitudinal GWAS, and the analysis could be completed within 1 day for each trait with known variances.

Several studies have reported that additive genetic, permanent environmental and phenotypic variances for milk production traits in dairy cattle vary with days in milk. Strabel et al. [21] reported the general patterns for the three variances in Polish Black and White cattle and showed that they were higher at the two extremes of lactation and lower in the middle part. Zavadilova et al. [22] reported a similar dynamic pattern in Czech Holstein cattle. However, El Faro et al. [23] showed that the estimated variances decreased continuously during the first lactation for Brazilian Caracu heifers, while Gebreyohannes et al. [24] observed a continuous increase in an Ethiopian dairy cattle population. In spite of these different results for various dairy cattle populations, the overall dynamic pattern reveals that the expression of genes and the influence of environment on phenotype are time-dependent. Acknowledging this fact and analyzing the data with dynamic models will help to better understand the genetic control of longitudinal traits.

Here, we presented the advantage of the Eigen decomposition approach for dealing with longitudinal GWAS for milk production traits in Chinese Holstein. The longitudinal GWAS method can be also applied to other kinds of longitudinal data, such as daily gain in pig and egg weight in chicken. Furthermore, Eigen decomposition can be generalized to different linear mixed models. For a GWAS model with only one variance component other than the error variance, we can expedite the speed of the estimation of variance components to enhance the computational efficiency as in EMMA (efficient mixed-model association) [8]. If we include additional variance components, such as non-additive genetic variances and permanent environmental variance, into the GWAS model, a two-step strategy as described in our study can be used. First, the variance components are estimated only once in the model not including the SNP effect, and second Eigen decomposition of merged variances other than independent error variances is applied to improve the performance of GWAS in a more general way.

Biological curve parameters of Wilmink polynomials can also be used in the longitudinal analysis of milk production traits [14]. However, the Wilmink model can result in a convergence problem in the estimation of variance components due to higher correlations between the estimated regression coefficients. Legendre polynomials can reduce the correlations and make no assumption about the shape of the curve; therefore, they are popular in dairy cattle breeding [25, 26]. The orders of Legendre polynomials will influence the performance of our longitudinal GWAS model. Akaike information criterion (AIC) or cross-validation are usually used to determine the orders of these functions in other longitudinal studies [18, 27]. However, it is computationally expensive to estimate the variances of a random regression model with a dense marker-derived genomic relationship matrix. In this study, we applied fourth-order Legendre polynomials to all time-dependent effects, which is preferred in genetic evaluation methods for dairy cattle [28, 29]. The simulation study based on the real dairy cattle dataset indicated that our longitudinal GWAS model with fourth-order Legendre polynomials could control the type I error in our study population. However, this is not a universal principle, and the optimal orders of the Legendre polynomials should be estimated for other longitudinal data.

Jiang et al. [3] performed GWAS for milk production traits in Chinese Holstein using EBV for 2093 cows that were estimated based on a multiple trait random regression test-day model. In general, our method identified fewer significant SNPs for the corresponding traits. In addition to different statistical methods, the main reason is that the marker-derived genomic relationship matrix was used to further control the type I error. Furthermore, the fact that similar and narrower peak regions were obtained in our study compared to the previous one [3] validates this method and indicates that it could be a more accurate approach for future studies. We identified 84 significant SNPs for three traits, of which 65 were located within known QTL regions. Among the remaining SNPs, six significant associations for FP were located within 110 kb of QTL reported by Lehnert et al. [30], and this region can represent a new potential QTL for FP. Thirteen additional SNPs for PP were 4 to 1724 kb away from the nearest QTL, and we extended the QTL region for PP.

## Conclusions

In summary, we propose a rapid GWAS method for longitudinal data. In a situation where variances are known, the GWA analysis could be completed within one day for thousands of individuals with tens of thousands of records genotyped with the 70 K chip. We applied our method to the analysis of milk production traits in Chinese Holstein. The performance with simulation and real data showed that our method could control the type I error. We located 84 significant SNPs by our methods of which 65 were within reported QTL regions. Nineteen new SNPs were identified, which could be new candidate QTN for milk production traits in Chinese Holstein.

## Additional file

**Additional file 1: Table S1.** Genome-wise significant SNPs and the corresponding information for genes within 100 kb and the nearest QTL with MY, FP and PP.
